# Monte Carlo Simulations of Polymer Collapse in an Explicit Solvent of Varying Quality

**DOI:** 10.3390/polym17070978

**Published:** 2025-04-03

**Authors:** Piotr Polanowski, Andrzej Sikorski

**Affiliations:** 1 Department of Molecular Physics, Technical University of Łódź, 90-924 Łódź, Poland; piotr.polanowski@p.lodz.pl; 2 Faculty of Chemistry, University of Warsaw, Pasteura 1, 02-093 Warsaw, Poland

**Keywords:** coil-to-globule transition, lattice models, Monte Carlo method, polymer solutions

## Abstract

The behavior of a single homopolymer chain in an explicit solvent in a wide range of poor and good solvents was investigated. For this purpose, a two-dimensional coarse-grained model based on a triangular lattice was used. Simulations were carried out by the Monte Carlo method using the Cooperative Motion Algorithm to study high-density systems. The scaling relations of the parameters describing the phase transitions of the chain were determined. For systems with polymer–solvent attraction, significant changes in chain size and shape were observed. This was associated with the mechanism of chain penetration by solvents and the formation of structures via a mechanism called ‘Bridging-Induced Attraction’, similar to those discovered for three dimensions.

## 1. Introduction

The effect of solvent quality on the conformation of a macromolecule and the coil–globule transition in polymer chains in solution has been a subject of research for many years, both experimental and theoretical. Experiments were carried out to study the conditions under which this phenomenon occurs and to determine the parameters of this transition, using various techniques such as light scattering, viscometry and NMR [1,2,3,4,5,6,7,8,9,10,11,12,13,14,15,16,17,18]. Theoretical studies of various models of a macromolecule in solution have been carried out primarily using mean-field theory and scaling analysis [19,20,21,22,23,24,25,26,27,28,29,30,31]. Numerous polymer chain models have also been studied by computer simulation techniques, using Monte Carlo methods [25,32,33,34,35,36,37,38,39,40,41,42,43], Molecular Dynamics [38,44,45,46,47,48,49,50], Brownian Dynamics [51,52,53,54,55] and Dissipative Particle Dynamics [56,57,58]. For homopolymers, decreasing the temperature and therefore the quality of the solvent causes a transition from a loose coil to a densely packed globule, and then crystallization takes place. These two transitions correspond to gas–liquid and liquid–solid transitions in simple liquids [4,59,60,61,62,63]. Simulation studies of chain collapse in the presence of explicit solvent molecules are much less numerous due to computational difficulties. Longer chains placed in sufficiently poor solvents seemed to collapse through the crushed globule into the equilibrium globule [56]. The effect of solvent molecule size on the globule–globule transition [21] and the effect of the presence of other objects in the system (crowding), which shifts the globule–globule transition toward higher temperatures, have also been studied [64].

Two-dimensional models, which formally can be thought of as representations of strongly adsorbed macromolecules and very thin polymer films, are particularly difficult for simulation studies because of the stronger effect of excluded volumes (not to mention the problems of designing efficient and correct simulation algorithms). Chain collapse in two dimensions has also been the subject of theoretical studies and simulations. Theories have shown that the collapse transition is second-order, using scaling analysis [65] and determining the exact partition function for short chains [66,67]. Monte Carlo simulations have also been performed, confirming that the coil–globule transition is second-order, while the crystal–globule transition is first-order, and providing an analysis of critical exponents [35,66,67,68,69,70,71,72,73,74,75,76,77]. The molten globule state has also been found for two-dimensional systems [77]. Two-dimensional polymer systems can thus adopt crystal, disordered globule and swollen coil states. Recently, the position of the theta temperature, the globule–globule transition and the critical exponents for the chain in a dense system, that is, when all the space is filled by the solvent, have been determined, while indicating the differences in structure between such a globule and the chain conformation in a dense polymer melt [78].

In this work, we studied the properties of single flexible homopolymer chains over a wide range of temperatures, but with a different set of interaction potentials than usual, namely, the repulsion of polymer and solvent. Therefore, we developed our work [78] by extending the study to the area of good solvents. In the current work, we focused on studying the differences in the properties of chain phase transitions in good and poor solvents. Such systems have been studied recently, but the simulations concerned three-dimensional systems [37,49,50,54,55], while we wanted to deal with exactly two-dimensional systems. Moreover, the chains studied were not very long, and the volume concentrations in the system were rather low (the total volume concentration of polymer and solvent did not exceed one-third). This weakness of these calculations is crucial, especially since in the high-density systems we were studying, it is the interaction of solvent molecules in the chain that forces changes in its conformation. It is therefore worth investigating what the coil–globule transition will look like when the space is completely filled with solvent molecules. In the model, a coarse-grained representation of the chain was introduced and a lattice approximation (triangular lattice) was used. The chain was placed in a system completely filled with explicit solvent molecules. The Cooperative Motion Algorithm (CMA), which works efficiently in macromolecular systems at high densities and for long chains, was used for the simulations. We focused our study on scaling relationships near the coil–globule transition point, as well as static and scattering properties. In our previous work [78], we investigated the same polymer model in the case of a poor solvent, for which the chain–coil transition temperature and the position of the maximum in the specific heat curves were determined. The results obtained were generally in agreement with existing knowledge. In the current work, we compared the results of that model with the properties of the chain in a wide range of good solvents.

## 2. The Model and the Method

Due to the complexity of macromolecular systems, simplified models were used for simulations. A coarse-grained representation of polymer chains was chosen for this study because of the need to simulate systems containing only one chain but many solvent molecules. The simulations also had to be long enough, due to the rather long relaxation times of macromolecules, which are particularly evident in two-dimensional systems. Our goal was to study the coil–globule transition, a phenomenon occurring at the scale of the entire chain, which means that a low-resolution model was most appropriate here. The model homopolymer chain was made up of *N* identical beads, each corresponding to a certain number of chemical mers. It was also assumed that the solvent molecules were the same size as the polymer beads; atomic details were therefore omitted in the model. This allowed a further approximation, i.e., the discretization of space: all objects (polymer beads and solvent molecules) were placed at the nodes of a triangular lattice. This lattice was chosen because of its high coordination number (*z* = 6). Due to the adoption of the lattice model, it was possible to use simplified interaction potentials, which were in the form of a square well [78]. The interacting objects in the system were polymer beads (P) and solvent molecules (S), which meant that there were three possible interaction potentials in the system: *E_PP_*, *E_PS_* and *E_SS_*. In most theoretical considerations, it is assumed that the polymer beads interact with each other with an attractive potential (*E_PP_* < 0), and the other interaction potentials are ignored: *E_PS_* = *E_SS_* = 0. This set of potentials leads to a positive Flory–Huggins parameter, χ, defined as

(1) $$\chi =\frac{z}{kT}\left(E_{PS}-\frac{E_{PP}+E_{ss}}{2}\right)\;$$

where *z* is the lattice coordination number, *k* = 1 is the Boltzmann constant and *T* is the temperature. For this standard set of interaction parameters, we were dealing with the well-known chain collapse at lower temperatures (or in the poor solvent), as demonstrated by numerous theoretical works [19,20]. But we could have also used a different model with interactions specific to the poor solvent: *E_PP_* = *E_SS_* < 0, *E_PS_* = 0 [78], which led to positive values of *χ*, and this model was also examined. Besides this model of interactions, we could have also chosen a set of potentials, corresponding to a negative χ value, for example, *E_PP_* = *E_SS_* = 0, *E_PS_* < 0, so here we considered only polymer–solvent attractions. As mentioned above, this is a similar model to that studied a dozen years ago [37] and can be treated as a good solvent.

Since one of the goals of this work was to study various interactions of the dense macromolecule with the solvent, the CMA algorithm was used [79]. It allows a chain to be studied in a space completely filled with solvent molecules, while almost all other simulation algorithms at such densities become completely ineffective [78]. CMA uses the idea of the cooperative movement of objects along closed paths in a lattice, where a chain element can change position only if neighboring polymer beads or solvent molecules (located at adjacent lattice sites) change their positions simultaneously with it. The basis, therefore, lies in finding such paths and then moving the elements forming the path by one place in the lattice, as long as such movement is possible, i.e., the integrity of the chains is not violated as a result of the movement and the movement does not lead to a situation in which the polymer bonds are crossed. This procedure leads to closed loops with a wide distribution of lengths but without self-movement (a loop can repeatedly cross itself). The sum of the displacements of the elements involved in the regrouping loop is zero (a continuity condition). During these rearrangements, the chain undergoes conformational transformations but retains its length and all bonds between the beads. The algorithm can be described as follows: (1) we temporarily remove one randomly selected element (temporary vacancy = TV); (2) the direction of movement of the TV is chosen randomly from among the adjacent points; (3) the element located at the randomly selected location swaps places with the TV, i.e., the TV moves to the selected location (the TV cannot move in any direction, as the continuity of the chain would be violated, but only along the chain until it encounters the end of the chain or a loop. In either case, there will be an interchange between the TV and a given element); (4) after passing through the end of the chain, the TV can again choose the direction of travel randomly from among the possibilities; (5) the TV can move to a new chain. The Monte Carlo step corresponds to an attempt to move, on average, each molecular element in the system. A detailed description of the CMA algorithm and a discussion of its applicability are presented elsewhere, to which we refer the interested reader [79,80]. The chain was first relaxed in the athermal system (without interactions, with excluded volume only), and then interactions were turned on and the relaxation was carried out to equilibrium, that is, until quantities such as parameters describing the chain’s size, energy and specific heat became stable. Each equilibration lasted for 10^7^ CMA steps. Once the system reached the equilibrium, the production run begun and lasted 10^7^ steps. Data were collected during the production runs every 10^5^ steps. For a given chain length, simulations were repeated 8–10 times, starting from different initial configurations. The author of the code is P. Polanowski. Simulations were performed on a PC with an Intel Core™ i-7 -2600 CPU @ 3.40 GHz under Linux and 8 Gb of memory. A single simulation run lasted about one week.

## 3. Results and Discussion

### 3.1. Phase Transitions

Below, we present the properties of the two models of polymers in solvents under consideration and their comparison. We further measured the quality of the solvent using the expression *ε_PS_* − (*ε_PP_* + *ε_SS_*)/2, where *ε* = *E*/*kT*. This expression corresponds to *χ*/*z*, and its value varied between −2 and 2, broadly covering the range of good and poor solvents. It was assumed that the values of non-zero interaction potentials were equal to −1: in the poor solvent model, *E_PP_* = *E_SS_* = −1, and in the good solvent model, *E_PS_* = −1. The temperature is defined as *kT* = *z*/*χ*|*E_PS_* − (*E_PP_*+*E_SS_*)/2|. The chain length was varied between *N* = 16 and *N* = 512. The size of the Monte Carlo box was 256 × 256. The edge of this box was an order of magnitude larger than the size of the longest chain under consideration (2 < *R_g_*^2^ > ^1/2^ << *L*, where < *R_g_*^2^ > is the mean-square radius of gyration of the chain). Periodic boundary conditions were also introduced.

**Figure 1:**
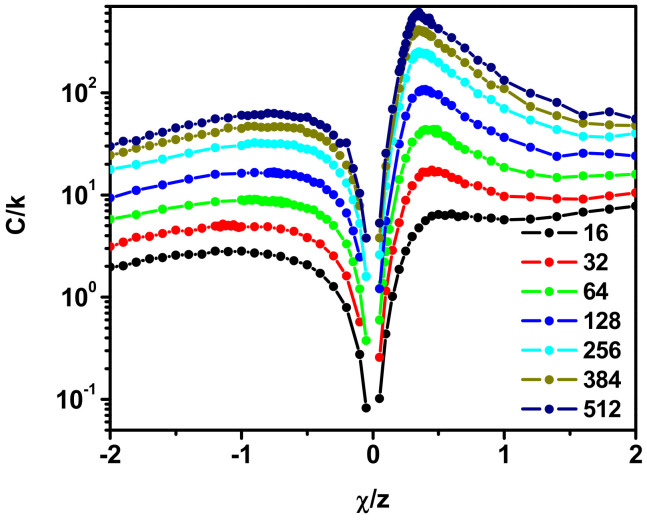
The heat capacity, *C*/*k*, as a function of *χ*/*z*. The lengths of the chain are given in the inset.

A convenient parameter that is sensitive to the occurrence of a phase transition in the system is the heat capacity. It can be determined directly from simulations using the expression

(2) $$C_{V}/k=\beta^{2}\left(\langle E^{2}\rangle -\langle E^{2}\rangle \right)\;$$

where *E* is the total energy of the system and *β* = 1/*kT*. Figure 1 shows the value of heat capacity as a function of the *χ*/*z* parameter. For positive *χ*/*z* values (hereafter referred to as the good solvent), we see that all *C_v_* curves have a similar shape with a well-defined maximum. The *C_v_* value increases with chain length, and the maximum on the *C_v_* curve, indicating the location of the phase transition, shifts toward larger *χ* values (which means that it increases with temperature) with increasing chain length. For the second model with *χ*/*z* < 0 (hereafter referred to as the poor solvent), we again see an increase in *C_v_* values with the number of segments in the chain. Maxima on the *C_v_* curves also appear, but the peaks are significantly less steep and wider compared to the model with the positive *χ* value. The maxima on the *C_v_* curves, as in the previous case, move toward higher temperatures with increasing chain length. The maxima in the *C_v_* curves on either side of *χ*/*z* = 0 indicate the presence of phase transitions, but the considerable difference in the shape of the curves suggests that these are completely different transitions.

**Figure 2:**
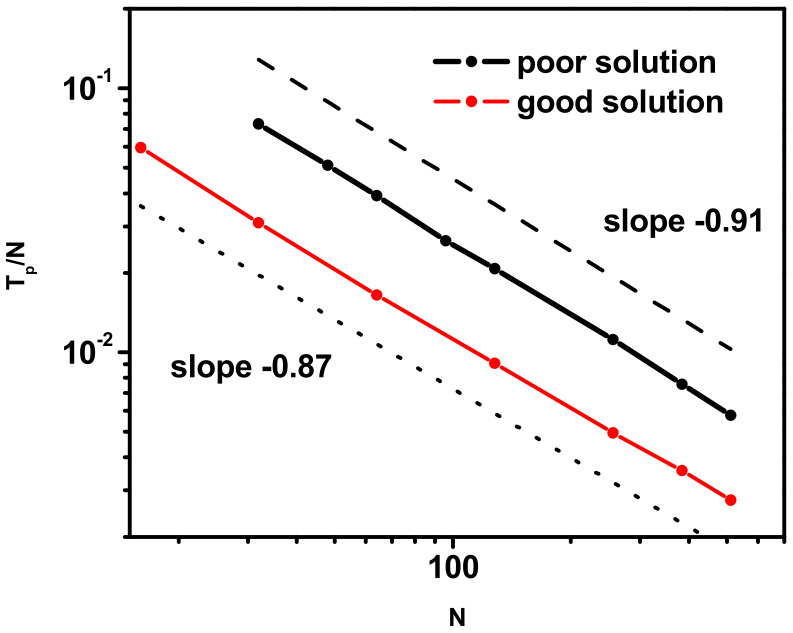
Scaling behavior of the phase transition reduced temperature, *T_p_*/*N*, as a function of chain length, *N*. See text for details.

One can also analyze the temperatures, *Tp*, corresponding to the phase transition indicated by maxima on the *C_V_*/*k* curves presented above in Figure 1. Figure 2 shows the reduced transition temperature, *T_p_*/*N*, versus the chain length, *N*, at the double logarithmic scale. This quantity in both cases, i.e., for *χ*/*z* < 0 and *χ*/*z* > 0, scales with the chain length, as *T_p_* ~ *N*^−α^, where *α* = −0.91 for *χ*/*z* > 0 and α = −0.87 for *χ*/*z* < 0.

**Figure 3:**
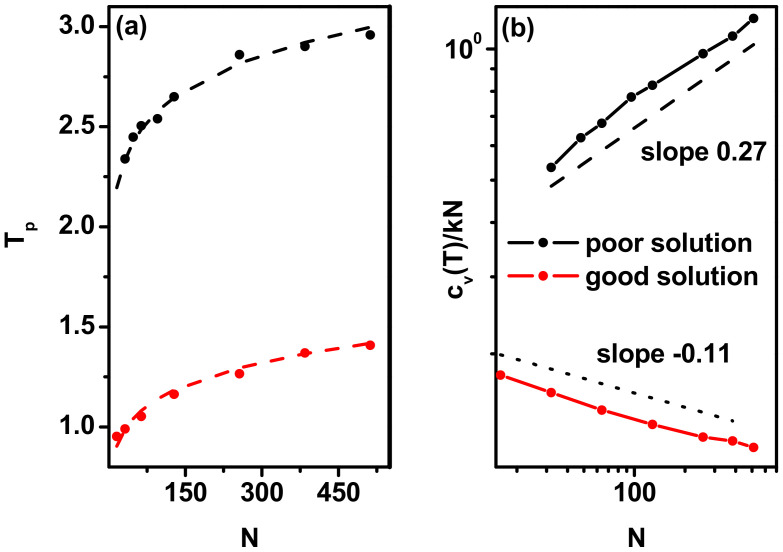
The transition temperature, *T_p_*, in cases of good and poor solvents obtained directly from simulation data (solid points) (**a**) and maximum values on the heat capacity curves in both cases as a function of chain length, *N* (**b**).

Thus, as mentioned above, the exact values of the phase transition temperature, *T_p_*, can be determined form the positions of the maxima on the *C_v_*(*T*) curves. In Figure 3, we present transition temperatures, *T_p_*, as a function of chain length, *N*. From the scaling relations discussed above, this dependency can be described as *T_p_* = 1.7 × *N*^0.09^ in case *χ*/*z* > 0 and *T_p_* = 0.63 × *N*^0.13^ for *χ*/*z* < 0. These relations are also marked in Figure 3a. It can be seen, indeed, that the temperature of *T_p_* increases with chain length, and this increase clearly decelerates for longer chains. The large differences in the pre-factors, 1.7 vs. 0.63, show, according to Figure 1, that in the good solvent the transition temperature is located considerably closer to *χ*/*z* = 0 than in the bad solvent. Figure 3b shows the changes in the maximum value of the reduced heat capacity (calculated per polymer bead, *C_v_*/*kN*) as a function of chain length. Here, the behavior of this parameter in the two models is different: it increases with the number of chain beads as *N*^0.27^ for *χ*/*z* > 0 (already determined in Ref. [68]) and decreases as *N*^−0.11^ for *χ*/*z* < 0.

### 3.2. Size and Shape of the Chain

**Figure 4:**
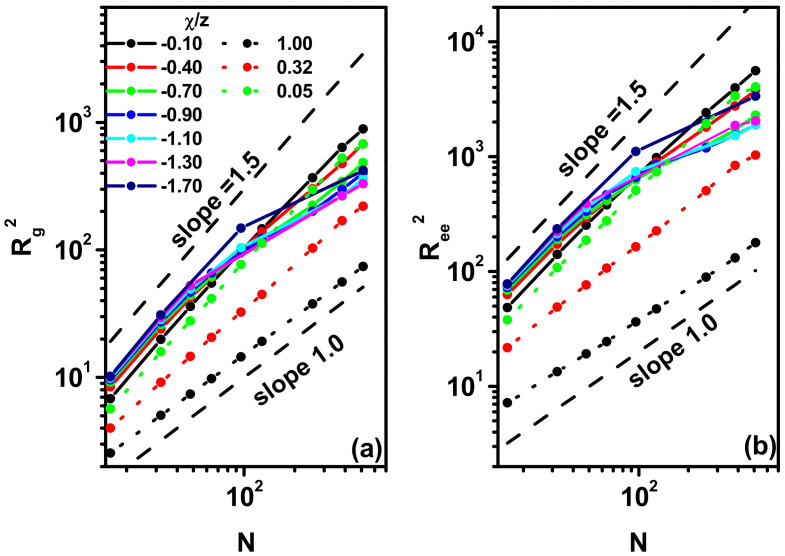
Log–log plot of the mean-square radius of gyration, < *R*_g_^2^ > (**a**), and the mean-square end-to-end distance, < *R*_ee_^2^ > (**b**), vs. chain length, *N*. The values of *χ*/*z* are given in the inset.

The next parameters considered are the mean-square end-to-end distance, < *R*_ee_^2^ >, and the mean-square radius of gyration, < *R*_g_^2^ >. These are parameters commonly used to describe chain size, defined as

(3) $$\langle R_{ee}^{2}\rangle =\langle {\left(r_{1}-r_{N}\right)}^{2}\rangle \;$$

(4) $$\langle R_{g}^{2}\rangle =\langle \frac{1}{N}\overset{N}{\underset{i=1}{\sum }}{\left(r_{i}-r_{cm}\right)}^{2}\rangle$$

where ***r***_1_ and ***r***_N_ are the coordinates of the two ends of the chain, ***r****_cm_* denotes the coordinates of the center of mass of the chain and ***r****_i_* denotes the coordinates of the i-th bead. Figure 4a,b present < *R*_g_^2^ > and < *R*_ee_^2^ > as a function of chain length, *N*. Let us first discuss the results obtained in the case of *χ*/*z* > 0 presented in Figure 4a,b as dotted lines. The scaling behavior < *R*_g_^2^ >, < *R*_ee_^2^ > ~ *N*^2ν^, is visible for all *χ*/*z* > 0: the exponent 2*ν* varies between values close to 3/2 and 1. Near *χ*/*z* = 0, the configuration of the polymer chain corresponds to a self-avoiding walk in two-dimensional systems and the exponent 2*ν* approaches theoretical value 3/2. For high *χ*/*z* close to 1, the polymer chains take the form of a compact disk and 2ν approaches the value 1. At the globule–coil phase transition temperature, *χ*/*z* ≈ 0.32, the polymer is in a critical state and theories predict that the scaling exponent takes the value 8/7 [30,31,81], which has been confirmed by most reliable simulations [35,78], although it should be remembered that older simulations delivered values for this exponent between 1.006 and 1.2 [35]. In general, the exponent 2*ν* weakly depends on the length of the chain. The situation changes significantly when *χ*/*z* becomes less than zero. In this case, both < *R*_g_^2^ > and < *R*_ee_^2^ > exhibit the scaling behavior for *χ*/*z* ≥ −0.1 only. For lower *χ*/*z* values, a clear deviation in the scaling behavior of the quantities characterizing the chain dimensions is visible. The reasons for this behavior will become clear after examining the behavior of the chain’s characteristics as a function of *χ*/*z* for individual chain lengths, as will be shown below.

**Figure 5:**
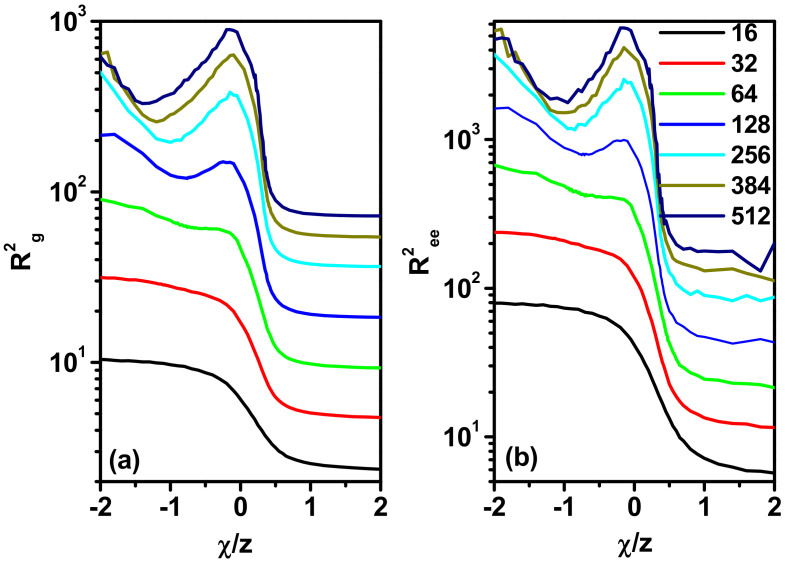
The mean-square radius of gyration, < *R*_g_^2^ > (**a**), and the mean-square end-to-end distance, < *R*_ee_^2^ > (**b**), as a function of *χ*/*z*. The lengths of the chain are given in the inset.

Figure 5a,b show the changes in both of these size parameters along with the value of *χ*/*z* for different chain lengths. It can be seen that both parameters behave very similarly, with the only difference being greater fluctuations in the value of < *R*_ee_^2^ > at low values of *χ*/*z*, which is understandable, since < *R*_g_^2^ > as the average value for the chain is more stable. It can be seen that for negative values of *χ*/*z*, we have a decrease in the size of the chain at temperatures corresponding to the *T_p_* values discussed above. The change becomes steeper as the number of beads in the polymer increases. This is the expected behavior, as it is characteristic of widely and deeply studied systems with *E_PS_* < 0 and *E_SS_* = *E_PP_* = 0. It can be seen that the introduction of an additional interacting solvent, *E_PP_* ≠ 0, does not qualitatively change the behavior of the chain in the *χ*/*z* > 0 region. In the *χ*/*z* < 0 region, the changes in chain size are much more interesting and surprising. Short chains (*N* = 16 and 32) behave differently here than long chains; however, it cannot be ruled out that all strings behave in the same way, and only this effect for short strings is weak. The difference is that with the increase in the strength of interactions, short chains decrease in size, similar to the model with *χ*/*z* > 0. For long chains, however, the course of change of < *R*_g_^2^ > and < *R*_ee_^2^ > is not monotonic. An initial decrease in size with temperature is followed by an increase in chain size, and further cooling leads to a decrease in size again. Models of three-dimensional systems containing explicit solvents at low concentrations indicated similar behavior of the chain radius of gyration, but the effect of non-monotonicity was significantly weaker there [37,49,50,54,55]. The reasons for this behavior will be discussed below.

We can also determine the shape of the chain from the analysis of the inertia tensor, *T_kl_*:

(5) $$T_{kl}=\langle \frac{1}{N}\overset{N}{\underset{i=1}{\sum }}\left(r_{ik}-r_{cm,k}\right)\left(r_{il}-r_{cm,l}\right)\rangle$$

where *k* and *l* are the coordinates x and y, *r_ik_* is the k-th coordinate of the position *r_i_* and *r_cm,k_* is the k-th coordinate of the chain’s center of mass. The eigenvalues of this tensor, *λ*_1_ and *λ*_2_, define for us the axes of the equivalent ellipse, which determines the shape of the chain [82,83].

**Figure 6:**
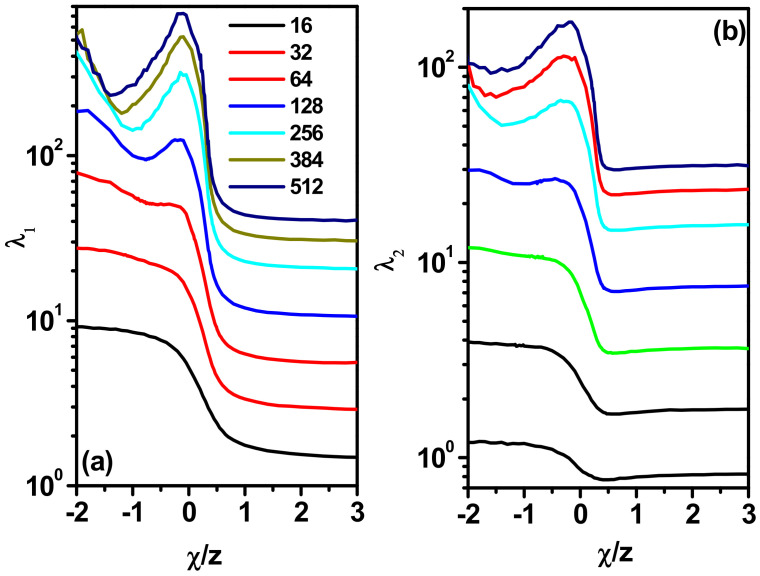
Plot of the mean values of *λ*_1_ (**a**) and *λ*_2_ (**b**) as a function of *χ*/*z*. The lengths of the chain are given in the inset.

Figure 6 shows the changes in both *λ*_1_ and *λ*_2_ along with the parameter *χ*/*z*. In this figure, we can see that the nature of the changes in *λ*_1_ and *λ*_2_ is exactly the same for the size of the chain, that is, < *R*_g_^2^ >. That is, we have a decrease in the values of both axes as the temperature decreases for *χ*/*z* > 0 and clear maxima appearing in the curves for *χ*/*z* < 0, while the changes in *λ*_1_ are more pronounced.

We can obtain additional information about the shape in a more convenient form from the analysis of the asphericity factor, *A*_2_. It is defined as follows [84,85]:

(6) $$A_{2}=\frac{{\langle \left(\lambda_{1}-\lambda_{2}\right)}^{2}\rangle }{{\langle \left(\lambda_{1}+\lambda_{2}\right)}^{2}\rangle }$$

**Figure 7:**
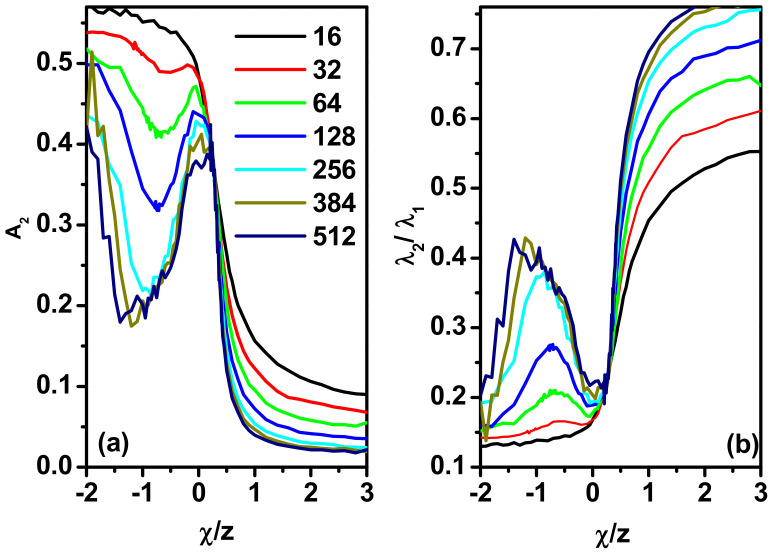
Asphericity *A*_2_ (**a**) and the *λ*_2_/*λ*_1_ ratio as a function of *χ*/*z* (**b**). The lengths of the chain are given in the inset.

This parameter takes a value of 0 for the disk and 1 for the rod. Figure 7a shows the change in the asphericity factor, *A*_2_, with the parameter *χ*/*z*, and Figure 7b shows the change in the ratio of the shape ellipsoid axes, *λ*_2_/*λ*_1_, with *χ*/*z*. Again, we see a monotonic decrease in the value of the *A*_2_ parameter with decreasing temperature for *χ*/*z* > 0 and a quick decrease in the asphericity for *χ*/z < 0. The ratio of the two *λ*_2_/*λ*_1_ axes shown in Figure 7b shows that for *χ*/z near zero, the chain has an ellipsoid shape because *λ*_1_ >> *λ*_2_. For *χ*/*z* > 0, when increasing its value, there is no stabilization of the chain’s shape but a temporary increase in *λ*_2_/*λ*_1_, that is, toward the disk.

**Figure 8:**
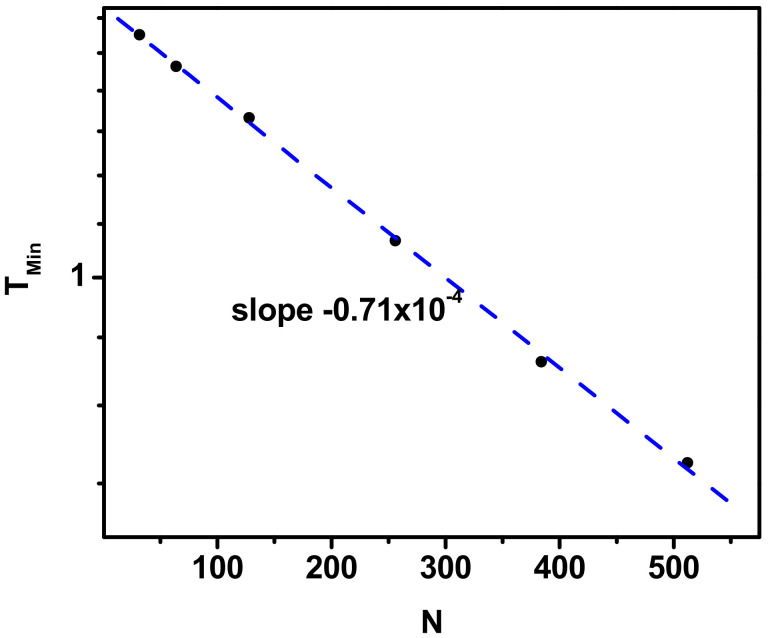
Position of minima on *A*_2_(*χ*/*z*) curves as a function of the chain length, *N*. Simulation results are shown as points, and the dashed line shows the fit (see text for details).

The dependence of the temperatures corresponding to the minima in the *A*_2_(*χ*/*z*) curves on the length of the chain is presented in Figure 8. It is clear that this characteristic temperature decreases with the length of the chain. From this figure, it can be seen that the change in the position of the point where the asphericity reaches a minimum value with the increase in the length of the chain has a semi-exponential character. Thus, it can be conjectured that this behavior indicates the entropic nature of the effect, since entropy increases with increasing chain length, which requires a shift toward lower temperatures and therefore a stronger interaction. On the other hand, the exponent is very small (*T_Min_* = 0.71 × 10^−4^), so the position of minima on *A*_2_(*χ*/*z*) curves depends very weakly on the length of the chain.

### 3.3. Scattering Properties

**Figure 9:**
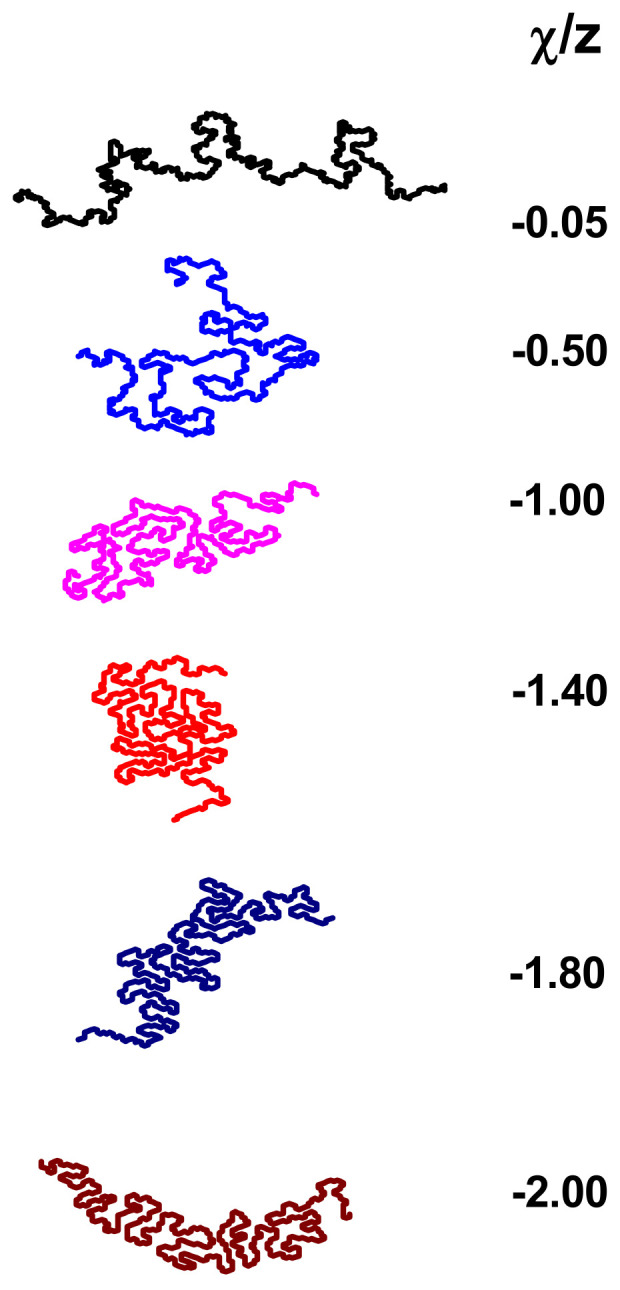
Snapshots of a polymer chain consisting of *N* = 512 beads for various negative values of *χ*/*z* indicated at the right.

Figure 9 visualizes the conformations of the long polymer chains (*N* = 512) tested for varying solvent quality. Since the structure of macromolecules in the negative *χ*/z region is well known, we present example conformations for positive *χ*/*z* values only. Looking at the conformations, one can see that as *χ*/*z* increases, the chain slowly begins to seem to collapse until it reaches *χ*/*z* = −1.40, that is, the point where the chain has the lowest value of asphericity. This effect seems to be due to the fact that with increasingly strong polymer–solvent interaction, different parts of the chain begin to be somehow bridged together by the solvent molecules. A similar mechanism called ‘Bridging-Induced Attraction’ was suggested for three-dimensional polymer chains in explicit solvents but at low densities [49,50,54,55]. In other words, the increase in the polymer–solvent potential leads to an increase in the number of polymer–solvent contacts, which must result in the transformation of a collapsed disk, where there are not too many contacts, into a more stretched form, as visible in Figure 9. This is because a single solvent molecule can effectively bridge two different parts of the chain together, resulting in something like a coil or at least something that looks like a coil. For stronger interactions (χ/z < −1.40), the chain loses the shape of a coil; however, its shorter elements still look bridged (for higher *χ*/*z*, the chains look like model illustrations of blobs).

**Figure 10:**
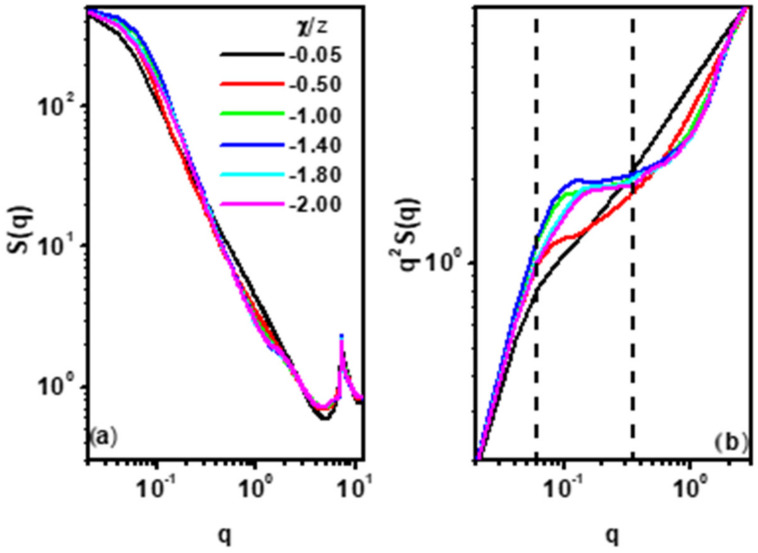
Single-chain scattering factor, *S*(*q*), for various values of *χ*/z (**a**) and the Kratky plot for various values of *χ*/*z* (**b**). The case of chain *N* = 512. Two vertical dashed lines mark the intermediate scaling regime.

Direct observation of single polymer chains in real experiments concerning two-dimensional polymer systems is not a simple matter. A very effective but rarely used technique that allows such observation is Fourier-transform fluorescence spectroscopy (FT-FS) [86,87]. In practice, this is a commonly used technique due to the simplicity of small-angle X-ray scattering (SAXS). Using this technique, we can obtain the so-called static structure factor, which tells us about the scattering intensity as a function of the wave vector, *q*:

(7) $$S\left(q\right)=\sum_{ij}\gamma \left(r\right)\frac{\sin\left(qr\right)}{qr}$$

where *γ*(*r*) is the intramolecular site–site correlation function of sites i and j separated by *r* = |**r**_i_ − **r**_j_|, defined as

(8) $$\gamma \left(r\right)=\frac{1}{N}\langle c(\vec{r_{i}})\cdot c\left(\vec{r_{j}}\right)\;\rangle$$

where *c* is a contrast operator, assuming values of 1 for the sites occupied by elements of the same chain and assuming 0 everywhere else. We can obtain *γ*(*r*) via Fourier transforms of the structure factor, *S*(*q*). Knowledge of this function allows us to draw conclusions about the chain topology. Figure 10a depicts the single-chain structure factor, *S*(*q*), as a function of the scatter vector, *q*, for the chain length *N* = 512, for various values of *χ*/*z*. A clearer picture can be obtained from Figure 10b, which shows the Kratky plot, i.e., *q*^2^*S*(*q*) as a function of *q*. This type of plot makes it much easier to determine the structure of a chain than the usual *S*(*q*) [88,89]. Special attention has to be paid to the area between the two vertical dashed lines, which correspond to the so-called intermediate (scaling) regime. In this area, plateaus are clearly formed in the *q*^2^*S*(*q*) curves. The plateau widens and rises as the value of *χ*/z decreases. But for *χ*/*z* > −1.40 in the considered *q* range, decreasing width and *q*^2^*S*(*q*) values of the plateau are observed in the area. The presence of a plateau indicates the formation of a more compact (disk-like) chain structure, but, as we can see, only in the limited range of *χ*/z. This behavior is related to the change in chain conformation observed in Figure 9. At the point *χ*/*z* = −1.40, the chain takes a shape most resembling a disk, which corresponds to the highest value of *q*^2^*S*(*q*), and then because a further decrease in *χ*/z leads to chain stretching, the value of *q*^2^*S*(*q*) decreases and a stretched but locally folded (bridged) chain appears.

**Figure 11:**
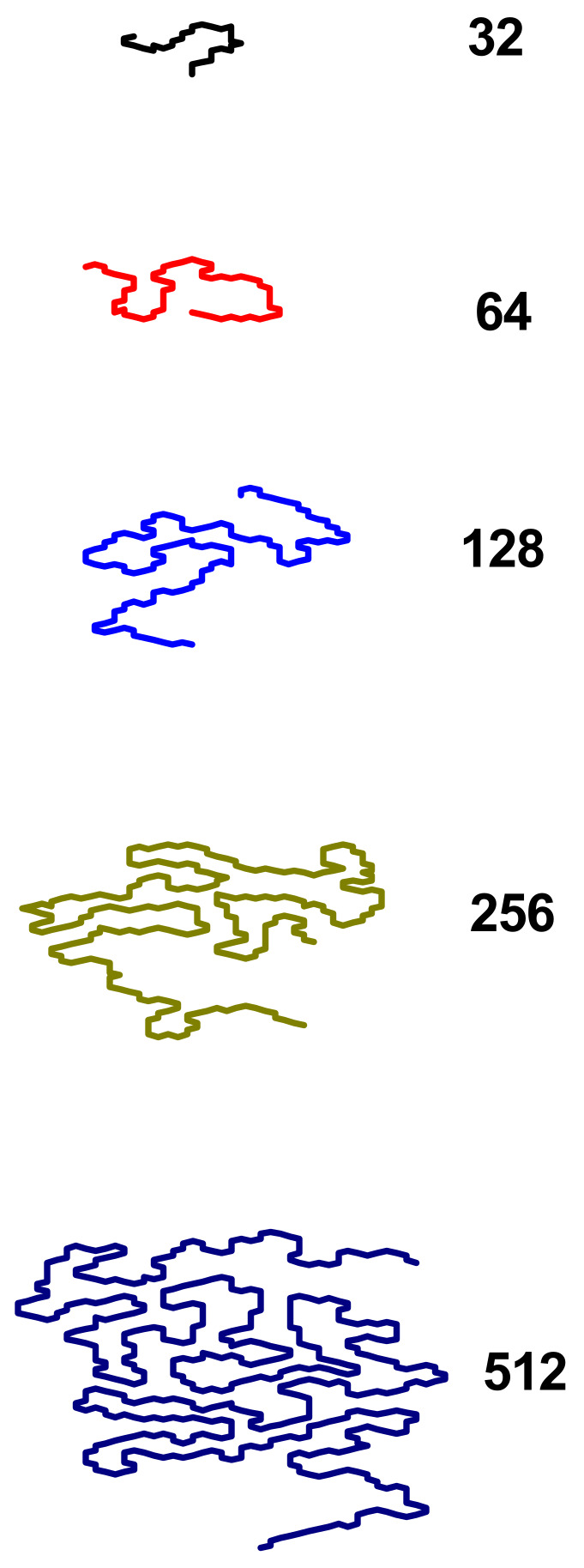
Snapshots of a polymer chain near the minimum value of asphericity for various chain lengths indicated at the right side.

The next visualization, presented in Figure 11, shows chain conformations for the parameter *χ*/*z* near the minimum of chain asphericity (i.e., for poor solvent conditions) for different lengths of the macromolecule. It can be seen that the longer the chain is, the more spherical its shape is, which is in complete agreement with the sequence of *A*_2_(*χ*/*z*) curves for the poor solvent presented in Figure 7a. Moreover, it turns out that the longer the chain, the more places there are where its fragments can be bundled by the solvent, maximizing the number of polymer–polymer and solvent–solvent contacts, and thus forming some kind of a disk. This can also explain the fact that as the chain length increases, the asphericity minima become deeper.

## 4. Conclusions

Simulations of a boundary-diluted two-dimensional polymer solution were carried out for two rarely used sets of interactions: polymer–polymer and solvent–solvent attraction (the Flory–Huggins parameter, *χ* > 0) and polymer–solvent attraction (*χ* < 0). A model of such systems based on a triangular lattice was used. The system was completely filled, that is, there were objects at all nodes of the network: beads of polymer or solvent. Due to the high density, simulations were carried out using the Cooperative Motion Algorithm.

It was shown that for a system where only polymer beads interact with each other and solvent molecules interact with each other (i.e., a system with a positive value for the *χ* parameter), a single-phase transition occurs, as in systems where only a polymer exists, as well as a polymer in an inert solvent. This transition involves the formation of a densely packed disk-like arrangement by the chain at lower temperatures. In a system where we have only polymer–solvent interactions (i.e., for negative *χ*), when the temperature increases, we observe an unexpected increase and decrease in the size of the chain, which is also associated with its more spherical shape. Similar and even more pronounced behavior was found for the chain shape expressed by the asphericity factor. The explanation for this non-monotonic behavior of the size and shape of the chain is the mechanism of chain penetration by the solvent. This is because solvent molecules interacting with the polymer bridge its fragments (a mechanism called ‘Bridging-Induced Attraction’) to form a pseudo-coil, and further worsening of the solvent leads to more extended conformations with shorter chain fragments bridged by the solvent. The influence of bridging is stronger that in three-dimensional systems.

## Data Availability

Dataset available on request from the authors.
